# Correction: Attenuation of experimental bronchopulmonary dysplasia by dimethyl fumarate

**DOI:** 10.1038/s41390-026-05146-6

**Published:** 2026-05-26

**Authors:** Fred Graumuller, Divya T. Rajendran, Yahui Li, Zhaohui Xiong, Candice Bui-Linh, Pragnya Das, Xiaoxin Chen, Vineet Bhandari

**Affiliations:** 1 Division of Neonatology, The Children’s Regional Hospital at Cooper/Cooper Medical School of Rowan University, Camden, NJ USA; 2 Division of Surgical Research, Department of Surgery, Cooper University Hospital/Cooper Medical School of Rowan University, Camden, NJ USA; 3 Coriell Institute for Medical Research, Camden, NJ USA

Correction to: *Pediatric Research* https://doi.org/10.1038/s41390-026-05039-8, published online 16 May 2026

In this article, the typographical mistake in the Y-axis of Fig. 2b has been corrected. It should be “Nrf-2 quantification” instead of “qualification”.

The original article has been corrected.

